# Mulberry Leaf Glutelin: Physicochemical, Functional, and Pancreatic Lipase Inhibitory Activity of Seven Varieties

**DOI:** 10.3390/foods14234004

**Published:** 2025-11-22

**Authors:** Hongyan Li, Dongjun He, Xiaomin Zhang, Zhenpeng Liu, Mingxi Li, Tianran Shen, Shuang Wei, Xiyang Wu, Chongzhen Sun

**Affiliations:** 1 School of Public Health, Guangdong Pharmaceutical University, Jianghai Avenue 283, Haizhu District, Guangzhou 510006, China; 2112241029@stu.gdpu.edu.cn (H.L.); 2112341015@stu.gdpu.edu.cn (X.Z.); 2112341024@stu.gdpu.edu.cn (Z.L.); limingxi613@163.com (M.L.); shentrgz@163.com (T.S.); 2 Department of Food Science and Engineering, Jinan University, Huangpu Road 601, Guangzhou 510632, China; 13202457231@163.com; 3 Guangzhou Customs Technology Center, Guangzhou 510623, China; weis200609@163.com

**Keywords:** mulberry leaf glutelin, ultrasound-assisted extraction, amino acid composition, techno-functional properties, pancreatic lipase inhibition

## Abstract

Mulberry leaf glutelin (MG), a nutrient-rich protein fraction from mulberry leaves, remains underutilized due to limited studies on its physicochemical functional properties and biological activities. In this study, seven varieties of MG (TSG, DSG, 109G, C1G, C2G, C3G, C4G) were evaluated for amino acid composition, secondary structure (FTIR), solubility, water-holding capacity (WHC), oil absorption capacity (OAC), foaming capacity (FC), foam stability (FS), emulsifying activity index (EAI), emulsion stability index (ESI), in vitro digestibility, and pancreatic lipase inhibitory activity (PLI). The results showed that MG contains four secondary structures and 17 amino acids, being rich in glutamic acid, aspartic acid, and leucine. C3G exhibited superior solubility (96.32%) at pH 10, while C4G showed optimal WHC (9.27 g/g), FC (73.75%), and FS (92.80%). TSG exhibited the highest OAC (9.58 g/g) and EAI (15.79 m^2^/g), and DSG demonstrated an excellent ESI (117.25 min), digestibility (88.17%), and PLI (70.54%). These findings provide valuable insights for the application of MG in food processing and innovation, enhancing its potential value for the food industry and human health.

## 1. Introduction

Plant leaf proteins are among the largest renewable protein resources globally, offering balanced nutrition and considerable economic potential [1]. The protein content of plant leaves directly influences their extraction yield [2]. Mulberry leaves are rich in nutrients, with a protein content ranging from 19% to 27%, underscoring their significant potential for exploitation in the food industry [3,4]. Modern nutritional science emphasizes that protein nutrition is largely driven by amino acids, and mulberry leaves have not only a high protein content but also a well-balanced amino acid profile, containing 17 amino acids, including 8 essential ones [5].

Despite the abundant mulberry leaf resources—characterized by extensive cultivation across Asia, Europe, and the Americas, where it is grown for silkworm feed, fruit production, and medicinal uses—their utilization rate remains extremely low, resulting in severe annual waste [1,4]. Studies on mulberry leaf proteins have mainly focused on two areas: the optimization of protein extraction and the analysis of its physicochemical properties [2,3] and the evaluation of its nutritional characteristics [5,6]. Current studies on the extraction and processing methods of mulberry leaf protein are diverse, including acid–base precipitation, salting-out, lactic acid fermentation, and foam separation [6]. Among these, acid–base precipitation is the most commonly used method in industrial applications due to its cost-effectiveness and high efficiency. Ultrasound-assisted extraction, as an auxiliary technique, can significantly enhance protein extraction yields and improve functional properties [7]. This is due to its ultrasonic effects, which can alter the medium’s morphology and structure. For instance, Wang et al. [8] optimized the functional properties of mulberry leaf protein using ultrasound-assisted extraction followed by a pH-shifting method to form composite protein nanoparticles. Li et al. [9] significantly increased the yield of mulberry leaf protein through multi-frequency ultrasound-assisted cellulase extraction.

Mulberry leaves contain four distinct protein fractions, with the following distribution of protein content: albumin > glutelin > globulin > prolamin. In our previous study, glutelin demonstrated the highest protein purity through Osborne fractionation [3]. While research on mulberry leaf protein has predominantly focused on albumin, studies on mulberry leaf glutelin remain scarce. However, glutelin, as a crucial protein fraction in plants, has been extensively studied for its physicochemical and functional properties across various plant species, such as rice glutelin [10], carob germ glutelin [11], wheat glutelin [12], and corn glutelin [13]. Recently, glutelin has been widely researched in animal feed, plant-based emulsifier [10], and glutelin-based films [14].

This study investigated seven varieties of commercial mulberry leaves, employing alkali extraction, acid precipitation, and ultrasound-assisted extraction techniques to isolate mulberry leaf glutelin. The physicochemical and functional properties and pancreatic lipase inhibition activity of these seven types of mulberry leaf-derived glutelins were evaluated to identify varietal differences. The findings aim to enrich glutelin varieties, provide a theoretical foundation for enhancing the functional properties of mulberry leaf glutelin, and help in developing functional foods derived from this high-quality protein source.

## 2. Materials and Methods

### 2.1. Materials

Seven varieties of mulberry leaves, namely Tangshi (TS), Da10 (DS), 109 (109), Caisang No. 1 (C1), Caisang No. 2 (C2), Caisang No. 3 (C3), and Caisang No. 4 (C4), were provided by the South China Sub-Nursery of National Mulberry Germplasm Resources (Guangzhou, China). Pepsin and trypsin were purchased from Solarbio Co., Ltd. (Beijing, China). Soy protein isolate (SPI) was purchased from Shanghai Yuan Ye Biotechnology Co., Ltd. (Shanghai, China). Pancreatic lipase and p-NPB were both purchased from Shanghai Maclin Biochemical Technology Co., Ltd. (Shanghai, China). The Bradford protein kit was from BBI LIFE SCIENCES. Sodium hydroxide, hydrochloric acid, and all other reagents were of analytical grade.

### 2.2. Preparation of Mulberry Leaf Glutelins

Protein fractions were extracted using the classic Osborne method with slight modifications based on the approach described by Sun [15]. In brief, mulberry leaf powder was first dissolved in deionized water (flour–solution ratio of 1:15), subjected to ultrasonic pulverization (400 w, 35 °C) for 15 min, and stirred for 120 min to maximize protein solubilization. Then, it was centrifuged at 8000× *g* for 15 min, and the precipitation was dispersed in a 0.05 mol/L NaOH solution (flour–solution ratio of 1:12), subjected to ultrasonic pulverization (400 w, 35 °C) for 15 min, and stirred for 120 min. The resulting suspension was then centrifuged at 8000× *g* for 15 min, and the pH of the extract was adjusted to the isoelectric point (pH 4) using HCl to precipitate the proteins. Then, the seven varieties of mulberry leaf glutelins (labeled as TSG, DSG, 109G, C1G, C2G, C3G, C4G) were subsequently obtained through centrifugation (12,000× *g*, 20 min); neutralization (pH 7.0); dialysis (with a 4.5 kDa cut-off) for 8–12 cycles until the conductivity was close to that of distilled water; and freeze-drying for 36 h. The extraction yield and protein purity were also determined. The total protein content in glutelin was quantified using the Bradford method, with BSA as the reference standard [16]. The yield of MG was calculated as follows:

$$Protein\;yield\;(\%)=\frac{Weight\;of\;lyohilized\;protein\;sample\;(g)}{Weight\;of\;mulberry\;leaf\;flour\;used\;for\;extraction\;(g)}\times 100\%$$

### 2.3. Amino Acid Analysis

The functional properties of proteins are largely influenced by their amino acid profile, sequence, arrangement, and composition [16]. According to the method for the determination of amino acid composition in GB/T 5009.124-2016 [17], mulberry leaf glutelins were hydrolyzed with 6M HCl for 24 h at 110 °C. After appropriate dilution, the samples were filtered through a 0.22 μm organic filter membrane and then analyzed by an amino acid autoanalyzer (Hitachi, Tokyo, Japan).

PDCAAS (Protein Digestibility-Corrected Amino Acid Scoring) was conducted using the protein digestibility values derived from the in vitro method [18], with the calculation incorporating both the Amino Acid Score (AAS) and protein digestibility [19].

$$PDCAAS\left(\%\right)=AAS\times IVPD\times 100\%$$

$$AAS=\frac{Limitied\;amino\;acid\;content\;in\;glutelin}{Same\;amino\;acid\;in\;reference\;protein}$$

### 2.4. Scanning Electron Microscopy

Lyophilized MG was coated with a thin layer of gold and analyzed using a scanning electron microscope (Ultra 55, Carl Zeiss AG, Oberkochen, Germany) to examine its microstructure. This observation was conducted at an acceleration voltage of 6 kV with a magnification of 3000× and 10,000× [20].

### 2.5. Fourier Transform Infrared Spectroscopy (FTIR)

Fourier transform infrared spectroscopy (FTIR) (Nicolet iS50, Thermo Scientific, Waltham, MA, USA) was employed to record the infrared spectra of the protein samples within the wavenumber range of 4000–500 cm^−1^ [21], and Origin 2021 was used to draw them. The FTIR spectra revealed characteristic peaks that provided valuable information about the protein’s functional groups. Furthermore, we examined the amide I region (1700–1600 cm^−1^) in detail to determine the protein’s secondary structure composition. This analysis was performed through peak deconvolution using PeakFit software (version 4.12).

### 2.6. Functional Properties of Different Mulberry Leaf Glutelins

#### 2.6.1. Protein Solubility

Protein solubility is a key physicochemical property that significantly impacts functional characteristics, including gelation, emulsification, and foaming capacity. Our experimental framework was adapted from Calderón-Chiu et al.’s isoelectric precipitation protocol [22]. Briefly, soy protein isolate (SPI) and mulberry glutelin (MG) were separately dissolved in distilled water to prepare 0.1% (*w*/*v*) protein solutions. The pH of each solution was then adjusted to 2.0, 4.0, 6.0, 8.0, and 10.0 using 1 M HCl or 1 M NaOH. After pH adjustment, the samples were shaken at 25 °C for 30 min to ensure proper equilibration. Next, the mixtures were centrifuged at 7500× *g* for 15 min to remove insoluble material. Finally, the protein concentration in the supernatant was determined using the Bradford assay, with bovine serum albumin (BSA) serving as the standard reference.

$$Protein\;solubility\;(\%)=\frac{supernatant\;protein\;content}{total\;protein\;content\;with\;1\;M\;NaOH\;treatment}\times 100\%$$

#### 2.6.2. Water-Holding Capability (WHC) and Oil Absorption Capability (OAC)

The determination of water-holding capacity (WHC) was slightly modified based on the method described by Aderinola [23]. Briefly, SPI and MG were dissolved in distilled water and vortexed for 1 min, followed by incubation at 25 °C for 30 min. After centrifugation at 5600× *g* for 25 min, we carefully drained the supernatant at a 45° angle for 10 min. WHC was then calculated as grams of retained water per gram of protein (g/g), with three replicates per sample.

The oil absorption capacity (OAC) was assessed according to the method of Li et al. [24], with slight modifications. Briefly, test suspensions were prepared by homogenizing 50 mg protein aliquots (SPI/MG) with 1 g canola oil (1:20 *w*/*w*) using vortex homogenization (30 s, Genie 2™ mixer). Following thermodynamic equilibration (25 °C, 30 min), centrifugal phase separation was conducted at 4000 rpm for 30 min. Post-centrifugal drainage involved controlled oil decantation at 45° inclination for 20 min to minimize capillary action retention. OAC was expressed as grams of absorbed oil per gram of protein (g/g), with triplicate measurements.

#### 2.6.3. Foaming Capability (FC) and Foam Stability (FS)

Foaming capability and foam stability were measured with slight modifications based on the experiments of Rawiwan [25]. A 0.5% (*w*/*v*) solution of SPI and MG was prepared, mixed, and homogenized at 15,000 rpm for 3 min. Foam volumes were recorded immediately after homogenization and again after 15 min at room temperature (25 °C). Foaming capacity (FC) and foam stability (FS) were calculated using the following equations:

$$FC\;(\%)=\frac{V_{1}-V_{0}}{V_{0}}\times 100\%$$

$$FS\;(\%)=\frac{V_{t}}{V_{0}}\times 100\%$$

where *V*_0_ is the initial volume before whipping, *V*_1_ is the volume right after whipping, and *V*_t_ is the final volume after standing for 15 min.

#### 2.6.4. Emulsifying Activity Index (EAI) and Emulsion Stability Index (ESI)

The emulsification capacity of mulberry glutelin (MG) variants was characterized through interfacial adsorption kinetics and colloidal stability metrics, following established protocols [26] with critical methodological refinements. Protein dispersions (0.1% *w*/*v* in 10 mM phosphate buffer, pH 7.0) were homogenized with canola oil at a 1:3 oil-to-aqueous phase ratio using a T25 digital Ultra-Turrax homogenizer (13,000 rpm, 60 ± 2 s) to induce emulsion formation. Immediate post-homogenization sampling from the bottom phase of the centrifuge tube (50 μL aliquot) ensured the exclusion of creaming-layer interference, with subsequent dilution in 0.1% SDS solution (1:100 *v*/*v*) to stabilize droplet dispersion prior to spectrophotometric analysis at 500 nm (UV-1800, Shimadzu, Kyoto, Japan). Temporal stability was quantified through repeated measurements at 10 min intervals, capturing phase separation dynamics under gravitational stress. The emulsifying activity index (EAI) and emulsion stability index (ESI) for each glutelin were calculated using the following formulas:

$$EAI\;(m^{2}/g)=\frac{2\times 2.303\times A_{0}\times N}{C\times \phi \times 10000}$$

$$ESI\;(min)=\frac{A_{0}\times ∆t}{A_{0}-A_{10}}$$

where N represents the dilution factor (100), C represents the protein concentration (0.001 g/mL), ϕ is the oil phase volume fraction of the emulsion (0.25), ∆t refers to the settling time (10 min), and $A_{0}$ and $A_{10}$ correspond to the absorbance measured at 0 and 10 min, respectively.

### 2.7. In Vitro Protein Digestibility (IVPD)

The INFOGEST digestion model described by Brodkorb [27] was slightly modified. MG (0.1 g) was mixed with 1 mL of simulated salivary fluid (SSF) at pH 7.0, representing oral digestive fluid, and incubated at 37 °C with shaking at 60 rpm for 2 min. The resulting oral digestion mixture was then combined with 1 mL of gastric electrolyte containing pepsin (2000 U/L), adjusted to pH 3.0, and incubated in a thermostatic water bath shaker at 37 °C for 2 h at 60 rpm. Subsequently, 2 mL of intestinal electrolyte containing trypsin (100 U/L) and bile salts (10 mM) was added to the gastric digestion product, the pH was adjusted to 7.0, and the mixture was oscillated at 60 rpm for 2 h at 37 °C. At the end of the digestion process, the samples were immediately cooled in an ice bath for 10 min and centrifuged at 4000 rpm for 15 min, and then the protein content was measured by the Bradford method, with bovine serum albumin (BSA) serving as the standard reference. The in vitro protein digestibility (IVPD) was calculated as follows:

$$IVPD\left(\%\right)=\frac{N_{0}-N_{t}}{N_{0}}\times 100\%$$

where *N*_0_ refers to the initial total protein content of the sample, and *N*_t_ denotes the final undigested protein content of the sample.

### 2.8. Determination of Pancreatic Lipase Inhibitory Activity

Pancreatic lipase inhibitory activity (PLI) was determined following the method of Chen et al. [28] with slight modifications. Simulated intestinal fluid (SIF) contained 10 mM bile salts and was prepared as described by Brodkorb et al. [27]. Protein samples were dissolved in ultrapure water to prepare the 5 mg/mL protein solution. Meanwhile, the 2 mg/mL p-nitrobenzoic acid colorimetric reagent and the 2 mg/mL pancreatic lipase solution were prepared by dissolving in SIF, centrifugation (4000× *g* for 10 min), and filtration (0.22 μm). A total of 50 μL of protein sample solution was mixed with 50 μL of pancreatic lipase solution, incubated at 37 °C for 10 min, then reacted with 50 μL p-NPB (2 mg/mL) for 25 min at 37 °C. Absorbance at 405 nm was measured, with controls (sample, blank, and blank control) using SIF or water substitutions. Orlistat served as the positive control. Absorbance values were recorded as A_s_ (sample), A_b_ (blank), A_sc_ (sample control), and A_bc_ (blank control).

$$PLI\left(\%\right)=\frac{\left(A_{b}-A_{bc}\right)-(A_{s}-A_{sc})}{A_{b}-A_{bc}}\times 100\%$$

*A*_b_ stands for the blank group (absorbance of the mixture of water and enzyme solution)

*A*_bc_ represents the blank control group (absorbance of the mixture of water and simulated intestinal fluid)

*A*_s_ represents the sample group (absorbance of the mixture of sample and enzyme solution)

*A*_sc_ represents the sample control group (absorbance of the mixture of sample and water)

### 2.9. Statistical Analysis

All experiments were repeated 3 times, and SPSS (Version 22) software was used for data analysis. The results were expressed as the mean ± standard deviation. Figures were plotted by Prism (Version 10) and Origin 2021 software.

## 3. Results and Discussion

### 3.1. Extraction of Seven Different Varieties of Mulberry Leaf Glutelins

#### 3.1.1. Protein Yield and Content

**Figure 1:**
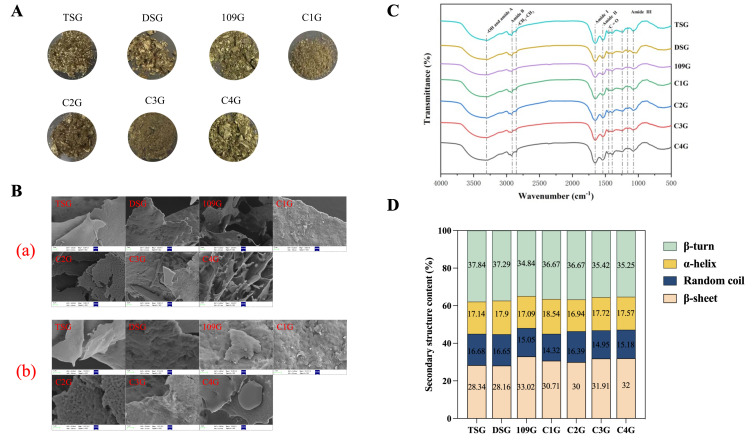
The structural characteristics of mulberry leaf glutelin. (**A**) Freeze-dried flour of seven kinds of mulberry leaf glutelin. (**B**) SEM images of different mulberry leaf glutelins at a magnification of 3000× (**a**) and 10,000× (**b**). (**C**) The Fourier transform infrared spectroscopy of seven mulberry leaf glutelins. (**D**) The secondary structure of each leaf glutelin.

The protein extraction rate is primarily influenced by the protein content of mulberry leaves and the extraction technique employed. Ultrasound-assisted extraction, as an auxiliary method, has been shown to significantly enhance protein yield and improve techno-functional properties [29]. In this study, mulberry leaf glutelins (MGs) were extracted using an alkaline solubilization and acid precipitation method combined with ultrasonic assistance, based on our previous work [3]. The appearance of the seven kinds of mulberry leaf glutelins is shown in Figure 1A. The extraction yield of the seven types of mulberry leaf glutelins ranged from 3.78% to 4.73%, with DSG exhibiting the highest yield at 4.73% (Table 1). However, this yield was lower than that reported for mulberry leaf albumin, which may be attributed to the inherently lower content of glutelin compared to albumin in mulberry leaves [3]. The alkaline extraction method coupled with the isoelectric precipitation methodology yielded mulberry glutelin isolates with purity levels spanning 53.59–66.36% (*w*/*w*). Among the fractions, C4G exhibited the highest purification efficiency (66.36 ± 0.83%), though it remained 12.4% below the values reported by Sun et al. [3], potentially attributable to interspecific variations in leaf source materials or residual non-protein nitrogen compounds.

Importantly, this extraction approach effectively preserved the bioactivity of the proteins by avoiding heat treatment and the use of organic solvents. Employing only NaOH as the extraction solvent, the method is environmentally friendly, sustainable, and non-polluting. Moreover, it demonstrated good reproducibility, cost-effectiveness, and operational efficiency, indicating its suitability for large-scale industrial application in protein extraction.

#### 3.1.2. Amino Acid Content and Composition

The amino acid profiling of mulberry leaf glutelins (MGs) revealed a comprehensive composition comprising 17 amino acids, including 9 essential amino acids (EAAs) and 8 non-essential amino acids (NEAAs) (Table 2). Notably, the total amino acid content of C4G (856.75 mg/g) and DSG (828.65 mg/g) significantly surpassed that of other varieties, with all MGs exhibiting a high abundance of glutamic acid, aspartic acid, and leucine. Among these, glutamic acid and aspartic acid are recognized as flavor-enhancing amino acids that critically improve the sensory attributes of food products, while leucine plays a pivotal regulatory role in muscle protein synthesis [30]. Further analysis demonstrated that C4G and DSG contained markedly higher levels of hydrophobic amino acids (HAAs) compared to other samples, suggesting the enhanced structural stability of their protein conformations [22]. Nutritionally, the aromatic amino acid (AAA) content of all MGs exceeded the minimum adult requirements established by the FAO/WHO [31]. TSG, DSG, and C4G exhibited particularly elevated sulfur-containing amino acid (SAA) levels, including methionine and cysteine. Methionine participates in methyl donor metabolism, while cysteine serves as a precursor for glutathione—a potent antioxidant peptide crucial for cellular oxidative stress defense [32]. The variability in SAA content may further influence functional properties such as solubility by modulating surface characteristics [33]. Of particular interest was the distribution of branched-chain amino acids (BCAAs; leucine, isoleucine, and valine), which can enhance the saltiness levels of vegetable soups while meeting clean-label requirements [34]. The total BCAA content in TSG, DSG, and C4G was significantly higher than that in other varieties (*p* < 0.05). In addition, the EAA/TAA (essential-to-total amino acid) and EAA/NEAA (essential-to-non-essential amino acid) ratios of all MGs exceeded the FAO/WHO reference values of 0.4 and 0.6, respectively [31], and were higher than those reported by Yan [5]. Beyond that, as shown in Table 3, the first limiting amino acid exhibits the lowest AAS value, and for all seven types of mulberry leaf glutelins, the first limiting amino acid is Met + Cys. In addition, as shown in Table 4, the score of the ratio coefficient (SRC) for MG samples ranged from 64.31 to 71.97, approaching that of soy protein isolate (SPI, 80), indicating a relatively high nutritional value. As reported in existing studies, in vivo and in vitro PDCAAS assay results demonstrate a high degree of similarity, indicating that in vitro characterization methods can partially substitute for in vivo protein quality evaluation [35]. Among the seven mulberry leaf protein fractions, DSG, TSG, and C4G exhibited prominent PDCAAS values. Specifically, DSG achieved the highest value of 68.30%, emerging as a component with the optimal nutritional value and possessing significant potential for further optimization or large-scale application. These findings collectively suggest that mulberry leaf glutelins are promising high-quality plant proteins with considerable nutritional potential.

#### 3.1.3. Surface Morphology

The microstructure of mulberry leaf glutelins was examined using field emission scanning electron microscopy (FE-SEM). As shown in Figure 1B, the surface morphologies of the seven glutelin samples were generally similar, though with subtle differences that may contribute to variations in their physicochemical and functional properties. Notably, C4G exhibited a typical honeycomb structure that was evenly distributed. This structure had good physical interception properties, which may contribute to C4G’s better water retention or oil absorption capabilities. TSG, C3G, and C4G exhibited relatively smooth surfaces punctuated by micropores, structurally analogous to the glutenin macropolymer architecture documented in wheat gluten fractions [37]. Prior studies have established a positive correlation between protein surface smoothness and enhanced solubility [38]. These microstructural observations validate the hypothesis that TSG, C3G, and C4G possess improved solubility profiles compared to other glutelin variants.

#### 3.1.4. FTIR Analysis

As shown in Figure 1C, the FTIR spectra of seven mulberry leaf glutelins exhibited consistent absorption patterns with characteristic peaks corresponding to specific functional groups. The broad absorption band at 3295 cm^−1^ (3100–3600 cm^−1^) was primarily assigned to the O-H stretching vibrations and N-H vibrations of amide A [39]. Two distinct regions were identified: the 2850–2950 cm^−1^ region showed peaks associated with amide B and aliphatic C-H stretching vibrations (-CH_2_/-CH_3_) from lipid components [40], while the 1200–1700 cm^−1^ fingerprint region encompassed overlapping signals from proteins, lipids, and polysaccharides [41].

Specifically, the prominent peak at 1650 cm^−1^ corresponds to the C=O stretching vibration of the amide I band, which is widely recognized as a sensitive indicator of the protein secondary structure [42]. Adjacent peaks at 1541 cm^−1^ and 1240 cm^−1^ were attributed to amide II (N-H bending/C-N stretching) and amide III (C-N stretching/N-H bending) bands, respectively [42]. Additional spectral features included the following: a lipid-specific peak at 1450 cm^−1^ (C=O stretching in COO^−^/COOH groups) [43], a weak peak at 1401 cm^−1^ suggesting phenolic constituents [43], and carbohydrate-associated vibrations at 1082 and 1165 cm^−1^ (C-C/C-O bonds) [44]. The deconvolution of the amide I region (1600–1700 cm^−1^) revealed eight component peaks corresponding to four secondary structure elements (Figure 1D): α-helix (1650–1660 cm^−1^), β-sheet (1600–1640 cm^−1^), β-turn (1660–1700 cm^−1^), and random coil (1640–1650 cm^−1^). All seven glutelins shared a similar structural profile dominated by β-sheet (28–32%), β-turn (34–38%), α-helix (16–19%), and random coil content (14–17%)—a pattern analogous to corn silk glutelins [21] but distinguished by the presence of detectable random coils. This structural divergence may originate from ultrasonic treatment, which is known to promote random coil formation [45].

Notably, Sample 109G contained the highest abundance of β-sheet structures (33.02%), which are closely associated with hydrophobic interactions in proteins, as subsequently confirmed by its poorest solubility [45]. Furthermore, the dense β-sheet architecture in 109G likely creates steric hindrance that restricts protease accessibility, thereby reducing in vitro digestibility [46].

### 3.2. Functional Properties of Seven Mulberry Leaf Glutelins

#### Solubility

**Figure 2:**
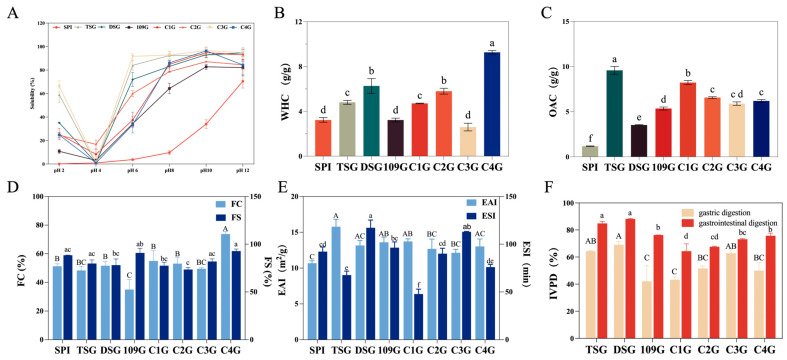
The functional characteristics of mulberry leaf glutelin. (**A**) The solubilities of seven mulberry leaf glutelins and SPI at pH 2.0, 4.0, 6.0, 8.0, 10.0, and 12.0. SPI, soy protein isolate, was used as the positive control. The WHC (**B**), OAC (**C**), FC and FS (**D**), EAI and ESI (**E**), and gastric and gastrointestinal digestibility (**F**) of seven mulberry leaf glutelins and SPI. Different letters indicate significant differences (*p* < 0.05).

Solubility is a fundamental physicochemical property that significantly influences the overall functionality of proteins, including their gelling behavior, emulsification efficiency, and foaming capacity. It is closely associated with the protein’s surface activity and is governed by multiple factors, such as the composition and spatial distribution of amino acid residues, molecular conformation, molecular weight, and structural flexibility. As shown in Figure 2A, the solubility of all seven glutelin samples followed a typical U-shaped trend with increasing pH, a pattern that aligns with the findings reported in the previous literature [47]. Among them, C3G demonstrated superior solubility across a wide pH range, reaching a maximum of 96.64% at pH 10. Notably, its solubility surpassed that of glutelins extracted from edible dock (*Rumex patientia* L. × *Rumex tianshanicus* A. Los) [16]. This enhanced solubility may be related to the lower sulfur-containing amino acid (SAA) and hydrophobic amino acid (HAA) content in C3G. Notably, the coexistence of high HAA content and favorable solubility in C4G and DSG can be explained by the spatial distribution of hydrophobic residues and the conformational characteristics of the proteins [48]. Specifically, these proteins may adopt a loose and flexible conformation, which reduces intermolecular hydrophobic aggregation, facilitates water molecule penetration, and thereby further enhances solubility (consistent with the inferences drawn from our previous SEM observations). As shown in Table 5, the solubility of all samples declined to a minimum at pH 4: SPI—0.92%; TSG—0.38%; DSG—1.15%; 109G—3.07%; C1G—8.29%; C2G—16.74%; C3G—1.60%; and C4G—0.94%. This may be attributed to the tendency of protein molecules to form large aggregates near their isoelectric point (pH 4), primarily through various non-covalent interactions [48]. Notably, mulberry leaf glutelins demonstrated superior solubility across a broad pH spectrum compared to commercial soy protein isolate (SPI), with their enhanced dispersibility indicating promising adaptability for innovative nutraceutical and functional food applications. This structural–function interplay—where the protein’s secondary conformation directly governs solubility dynamics—provides critical insights for rationally engineering food matrices with tailored techno-functional properties.

### 3.2.2. Water-Holding Capability (WHC) and Oil Absorption Capability (OAC)

The hydration performance of plant proteins significantly impacts food matrix engineering. Water-holding capacity (WHC) serves as a critical determinant of food matrix hydration states [49]. Notably, elevated WHC values (1.49–4.71 g/g) prove essential in viscosity-dependent food systems like gravies and artisanal baked goods where structural water management dictates product quality [24]. Our analysis revealed C4G glutelin as a hydration performance outlier, exhibiting exceptional WHC (9.27 g/g), as shown in Figure 2B. This phenomenon likely stems from its microstructural porosity and surface-exposed hydrophilic moieties that synergistically enhance water sequestration, surpassing commercial benchmarks like SPI and other botanical proteins by substantial margins [50].

Lipid interaction capabilities, quantified through oil absorption capacity (OAC), depend fundamentally on protein–lipid interfacial chemistry, and it is important to improve the taste, deep processing, and flavor of protein products [24]. As shown in Figure 2C, TSG exhibited the highest OAC value (9.58 g/g). Compared with SPI (1.17 g/g), MGs demonstrated significantly greater oil absorption capacities, ranging from 3.52 to 9.58 g/g. This enhanced OAC may be attributed to the frequent interaction between the protein’s non-polar side chains and the hydrocarbon chains of lipids, which promotes increased oil binding [48]. These differential functionalities position C4G and TSG as multi-modal food engineering tools—C4G for water-structured systems requiring moisture lockdown, TSG for lipid-intensive applications demanding flavor carriage and mouthfeel optimization.

#### 3.2.3. Foaming Properties

Foaming performance is generally influenced by factors such as protein concentration, solubility, pH [25], hydrophobicity, charged groups, molecular flexibility, and the number and distribution of polar groups [51]. As shown in Figure 2D, C4G exhibited the highest foaming capacity (FC) at 73.83%, producing dense and stable foam. This performance surpassed that of soy protein isolate (SPI, 51.25%) [52]. The superior foaming capacity of C4G may be attributed to its higher solubility, greater molecular flexibility, and enhanced exposure of hydrophobic residues, all of which facilitate rapid protein adsorption at the air–water interface and promote foam formation. In contrast, foam stability (FS) exhibited an inverse trend, except for SPI (88.42%) and C4G (92.80%), which maintained high stability, owing to the ability of proteins to act as foaming agents by adsorbing at the air–water interface and forming a protective layer, a process influenced by protein characteristics, solubility, concentration, and pH value [53]. Taken together, no significant differences were observed between SPI and C4G in either FC or FS, which suggest that C4G, similarly to SPI, possesses excellent foaming capacity and foam stability, underscoring its potential as an effective plant-based foaming agent for food applications. Such properties are particularly desirable in the formulation of whipped toppings, mousses, and aerated bakery products.

#### 3.2.4. Emulsifying Properties

The emulsifying capacity of food proteins is a pivotal attribute influencing their applicability in diverse food formulations, particularly those involving oil–water systems. These functionalities largely stem from the amphiphilic nature of proteins, which enables them to adsorb at the oil–water interface and reduce interfacial tension, thereby promoting emulsion formation and stability [26]. Emulsifying performance is typically evaluated by two principal indices: the emulsifying activity index (EAI), reflecting the ability to form emulsions, and the emulsifying stability index (ESI), which indicates resistance to coalescence over time. As shown in Figure 2E, all mulberry leaf glutelin samples demonstrated EAI values exceeding that of soy protein isolate (SPI, 10.68 m^2^/g), with TSG exhibiting the highest value (15.79 m^2^/g). This superior emulsifying activity may be attributed to the enhanced interfacial affinity of the glutelin fractions, likely due to a favorable amphiphilic balance that allows for rapid adsorption to the oil–water interface and an efficient reduction in interfacial tension [21]. Conversely, the ESI results revealed a distinct trend, wherein DSG showed the greatest emulsion stability (117.25 min). This could be linked to its surface-active profile, characterized by a higher proportion of hydrophobic amino acid residues that facilitate the formation of a robust interfacial film. Moreover, the abundance of charged residues—such as lysine, aspartic acid, and glutamic acid—may contribute to enhanced electrostatic repulsion and steric stabilization. In addition, the presence of elevated levels of polyphenols and polysaccharides in DSG may promote the assembly of protein–polyphenol–polysaccharide ternary complexes, structures known to reinforce the integrity and longevity of emulsions [7]. These results suggest that TSG is suitable for rapid emulsion formation, whereas DSG is more advantageous in stabilizing emulsions over time. Notably, although the total HAA (hydrophobic amino acid) content of C4G is the highest among the seven mulberry leaf glutelin varieties, its emulsifying properties are not ideal. This may be attributed to the fact that most of its HAA residues are buried inside the protein and cannot be fully exposed even under shear force during emulsification, ultimately resulting in decreased emulsifying activity and stability [26].

### 3.3. Digestibility

In vitro protein digestibility is a key parameter for assessing the nutritional quality of food proteins. Proteins from different sources exhibit distinct physicochemical and structural properties that influence both the rate and extent of proteolysis, as well as the nature of the resulting digestion products within the gastrointestinal tract [54]. As shown in Figure 2F, during the gastric digestion phase, TSG, DSG, and C3G exhibited relatively high digestibility values of 64.28%, 68.97%, and 62.78%, respectively—significantly higher than those of 109G (42.03%) and C1G (43.25%). This enhanced gastric digestibility may be attributed to the better solubility and more accessible surface structures of TSG, DSG, and C3G under acidic conditions, which facilitate enzyme–substrate interactions. During the intestinal phase, DSG (88.17%) demonstrated the highest gastrointestinal digestibility, surpassing both SPI (84.85%) and pea protein–wheat gluten [55]. In vitro protein digestibility is influenced by several factors, including amino acid composition, the protein’s spatial structure, solubility, and the extent of protein aggregation. The superior gastric and intestinal digestibility observed in TSG and DSG may result from their more favorable tertiary structures for protease accessibility, higher protein content, better solubility, and lower levels of interfering non-protein compounds, all of which enhance the exposure of peptide bonds to enzymatic hydrolysis [56]. These findings suggest that TSG and DSG are promising plant-based protein sources with high nutritional bioavailability.

### 3.4. Pancreatic Lipase Inhibitory Activity

**Figure 3:**
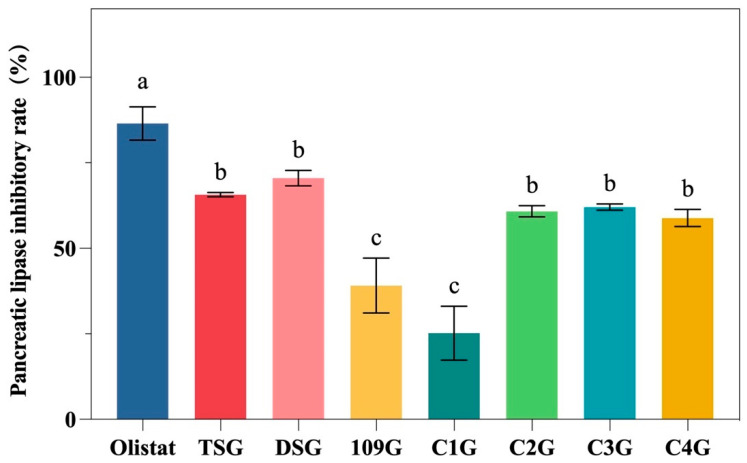
Pancreatic lipase inhibition rate of 7 kinds of mulberry leaf glutelin. Different letters indicate significant differences (*p* < 0.05).

Pancreatic lipase is primarily present in the intestine, where it facilitates the absorption of lipids by small intestinal epithelial cells through the hydrolysis of dietary fats [57]. Therefore, inhibiting pancreatic lipase activity can effectively reduce lipid absorption and help alleviate metabolic diseases such as obesity and non-alcoholic fatty liver disease (NAFLD). As shown in Figure 3, among the seven types of mulberry leaf glutelin, DSG exhibits the highest pancreatic lipase inhibition rate (70.54%), whereas 109G and C1G show the lowest inhibition rates at 39.11% and 25.69%, respectively. This disparity may be attributed to the significantly lower content of branched-chain amino acids in 109G and C1G compared to the other samples (*p* < 0.05). Interestingly, although C3G also has a relatively low content of branched-chain amino acids, it demonstrates a relatively high lipase inhibition rate. This could be due to its higher levels of polyphenols and polysaccharides, as mulberry leaf polysaccharides—being natural bioactive compounds—also exhibit inhibitory effects on pancreatic lipase, thereby enhancing the overall inhibition capacity of C3G [58]. While orlistat, a commonly used clinical pancreatic lipase inhibitor, achieves an inhibition rate as high as 86.50%, its long-term use is often associated with adverse effects such as diarrhea, oily stools, and the impaired absorption of fat-soluble vitamins. In contrast, mulberry leaf glutelin is a natural product derived from mulberry leaves, offering advantages in terms of accessibility, food safety, and overall health benefits. Although its lipase inhibition rate is slightly lower than that of orlistat, its safety, sustainability, and multi-functional bioactivity make it a promising candidate for the prevention and treatment of obesity, NAFLD, and other lipid metabolism-related disorders.

## 4. Conclusions

In this study, we assessed the extraction yield, amino acid composition, physicochemical functional properties, and pancreatic lipase inhibitory activity of seven types of mulberry leaf glutelins. The seven kinds of mulberry leaf glutelins were extracted using alkali dissolution and acid precipitation combined with ultrasonic-assisted extraction, each containing 17 amino acids, including 9 essential and 8 non-essential ones. The amino acid profiles were well-balanced, indicating that these glutelins are a high-quality protein source. Furthermore, FTIR analysis revealed consistent amide I, II, and III bands among samples, with β-sheets and β-turns as the dominant secondary structures. Despite structural similarities, notable differences were observed in functional properties. TSG showed excellent oil absorption and emulsifying capacity; DSG exhibited superior digestibility, emulsion stability, and lipase inhibition; C4G demonstrated strong water retention, foaming ability, and foam stability. These findings suggest that TSG and DSG have potential as nutritional supplements, with TSG suited for oil-based systems and DSG for lipid-lowering functional foods. Additionally, C4G may serve to enhance moisture retention in food applications.

Collectively, this study lays a theoretical groundwork for the application of mulberry leaf glutelin in food processing and functional food development while emphasizing that further investigations are required to fully exploit its inherent potential. In subsequent studies, we will focus on improving glutelin purification efficiency and systematically exploring structure–function relationships at the molecular level.

## Figures and Tables

**Table 1:** Protein yield and composition of 7 mulberry leaf glutelins.

Sample	Yield (%)	Protein Content (%)	Polysaccharide (g/100 g)	Polyphenol (g GAE/100 g)
TSG	4.64 ± 0.15 ^a^	61.36 ± 2.66 ^ab^	5.85 ± 0.08 ^f^	10.96 ± 0.48 ^d^
DSG	4.73 ± 0.24 ^a^	62.06 ± 2.10 ^ab^	13.21 ± 0.25 ^b^	19.32 ± 0.42 ^a^
109G	3.92 ± 0.04 ^b^	53.59 ± 1.83 ^c^	7.59 ± 0.25 ^e^	14.17 ± 1.82 ^c^
C1G	3.78 ± 0.11 ^b^	55.94 ± 2.99 ^bc^	11.13 ± 0.36 ^c^	15.64 ± 0.36 ^bc^
C2G	4.08 ± 0.17 ^ab^	59.59 ± 5.77 ^abc^	9.33 ± 0.40 ^d^	13.41 ± 0.13 ^cd^
C3G	4.07 ± 0.26 ^ab^	55.61 ± 1.90 ^bc^	19.55 ± 1.49 ^a^	17.59 ± 0.85 ^ab^
C4G	4.68 ± 0.27 ^a^	66.36 ± 5.95 ^a^	7.88 ± 0.09 ^e^	13.86 ± 2.47 ^c^

Notes: GAE, gallic acid equivalents. The values are expressed as the mean ± SD. Different letters within the same column indicate significant differences between samples (*p* < 0.05).

**Table 2:** Amino acid composition of 7 kinds of mulberry leaf glutelins (g/kg protein).

Protein Samples									FAO/WHO	
		TSG	DSG	109G	C1G	C2G	C3G	C4G	Child	Adult
non-essential	Asp	81.08 ± 1.45 ^b^	86.86 ± 0.55 ^a^	63.37 ± 0.03 ^d^	69.19 ± 1.14 ^c^	79.07 ± 1.02 ^b^	71.16 ± 0.69 ^c^	87.97 ± 0.23 ^a^		
amino acid	Ser	37.60 ± 0.68 ^ab^	38.24 ± 0.40 ^ab^	29.73 ± 0.48 ^b^	32.27 ± 0.53 ^b^	34.94 ± 1.91 ^ab^	31.78 ± 0.16 ^b^	39.34 ± 0.12 ^a^		
	Glu	109.43 ± 1.89 ^c^	114.91 ± 0.66 ^b^	80.07 ± 0.31 ^e^	87.49 ± 0.98 ^d^	105.12 ± 1.10 ^c^	89.85 ± 0.68 ^d^	120.35 ± 0.16 ^a^		
	Pro	30.42 ± 0.71 ^a^	34.98 ± 2.50 ^a^	24.84 ± 0.43 ^b^	27.12 ± 0.29 ^ab^	29.64 ± 0.44 ^ab^	27.58 ± 0.55 ^ab^	33.21 ± 0.11 ^a^		
	Gly	66.82 ± 1.17 ^b^	73.43 ± 0.31 ^a^	56.68 ± 0.15 ^c^	59.44 ± 0.35 ^c^	67.65 ± 1.80 ^b^	60.24 ± 0.17 ^c^	73.77 ± 0.04 ^a^		
	Ala	41.22 ± 0.29 ^a^	43.96 ± 0.02 ^a^	34.49 ± 0.01 ^b^	35.06 ± 0.89 ^b^	40.01 ± 2.45 ^ab^	37.17 ± 0.22 ^b^	44.78 ± 0.22 ^a^		
	Arg	45.75 ± 0.82 ^a^	44.35 ± 0.33 ^a^	33.05 ± 0.04 ^c^	34.30 ± 0.80 ^bc^	39.00 ± 1.36 ^b^	33.24 ± 0.29 ^c^	46.22 ± 0.30 ^a^		
	Tyr	34.85 ± 0.59 ^b^	37.51 ± 0.18 ^ab^	25.85 ± 0.19 ^c^	28.97 ± 0.89 ^c^	32.15 ± 0.68 ^bc^	28.75 ± 0.38 ^c^	40.51 ± 0.22 ^a^		
	Cys	3.26 ± 0.19 ^a^	3.11 ± 0.14 ^a^	1.94 ± 0.16 ^a^	2.80 ± 0.31 ^a^	2.98 ± 0.31 ^a^	1.86 ± 0.02 ^a^	3.23 ± 0.02 ^a^		
essential	Thr	38.10 ± 0.65 ^b^	43.14 ± 0.25 ^ab^	31.41 ± 0.11 ^c^	34.03 ± 0.62 ^bc^	39.13 ± 0.97 ^b^	33.68 ± 0.02 ^bc^	43.97 ± 0.12 ^a^	34	9
amino acid	Val	47.65 ± 0.70 ^a^	52.12 ± 0.07 ^a^	39.83 ± 0.28 ^c^	41.85 ± 0.01 ^bc^	46.62 ± 0.99 ^ab^	40.18 ± 0.29 ^c^	52.80 ± 0.29 ^a^	35	13
	Met	13.92 ± 0.25 ^a^	13.96 ± 0.14 ^a^	9.57 ± 0.00 ^b^	9.91 ± 0.59 ^b^	12.62 ± 0.65 ^b^	8.02 ± 0.02 ^b^	15.08 ± 0.04 ^a^		
	Ile	43.52 ± 0.72 ^a^	46.06 ± 0.24 ^a^	35.99 ± 0.10 ^b^	38.15 ± 0.34 ^b^	41.13 ± 1.08 ^a^	36.05 ± 0.32 ^ab^	46.24 ± 0.42 ^a^	28	13
	Leu	80.12 ± 1.14 ^a^	80.96 ± 0.18 ^a^	63.24 ± 0.25 ^c^	64.83 ± 0.13 ^c^	73.66 ± 1.46 ^b^	70.16 ± 0.56 ^b^	84.58 ± 0.08 ^a^	66	19
	Phe	50.08 ± 0.99 ^a^	51.21 ± 0.23 ^a^	39.87 ± 0.25 ^c^	42.03 ± 0.20 ^bc^	45.54 ± 0.26 ^b^	44.16 ± 0.43 ^bc^	52.77 ± 0.02 ^a^		
	Lys	40.47 ± 0.62 ^b^	40.33 ± 0.08 ^b^	41.46 ± 0.29 ^b^	41.58 ± 0.90 ^b^	42.73 ± 1.46 ^b^	43.61 ± 0.01 ^ab^	47.39 ± 0.04 ^a^	58	16
	His	23.81 ± 0.33 ^ab^	23.53 ± 0.08 ^ab^	19.17 ± 0.29 ^b^	19.80 ± 0.60 ^ab^	20.21 ± 0.37 ^ab^	20.74 ± 0.21 ^ab^	24.54 ± 0.05 ^a^	19	16
	ΣEAA ^1^	337.66 ± 5.41 ^c^	351.31 ± 1.27 ^b^	280.54 ± 1.14 ^f^	292.19 ± 0.57 ^e^	321.66 ± 1.80 ^d^	296.59 ± 0.57 ^e^	367.38 ± 0.58 ^a^		
	ΣNEAA ^2^	450.43 ± 7.79 ^b^	477.35 ± 0.56 ^a^	350.01 ± 0.63 ^e^	376.64 ± 3.26 ^d^	430.56 ± 6.88 ^c^	381.63 ± 3.12 ^d^	489.37 ± 0.81 ^a^		
	ΣTAA ^3^	788.09 ± 13.20 ^c^	828.65 ± 0.72 ^b^	630.55 ± 0.51 ^f^	668.83 ± 2.69 ^e^	752.21 ± 8.67 ^d^	678.23 ± 3.69 ^e^	856.75 ± 0.24 ^a^		
	ΣHAA ^4^	373.75 ± 5.98 ^b^	396.67 ± 1.31 ^a^	304.50 ± 0.94 ^e^	318.40 ± 1.14 ^d^	356.88 ± 3.69 ^c^	323.55 ± 1.33 ^d^	403.24 ± 0.24 ^a^		
	ΣBCAA ^5^	171.28 ± 2.56 ^c^	179.13 ± 0.49 ^b^	139.06 ± 0.43 ^f^	144.84 ± 0.47 ^e^	161.42 ± 0.61 ^d^	146.38 ± 0.05 ^e^	183.63 ± 0.62 ^a^		
	ΣSAA ^6^	17.19 ± 0.44 ^ab^	17.08 ± 0.01 ^ab^	11.51 ± 0.16 ^cd^	12.71 ± 0.28 ^c^	15.60 ± 0.96 ^b^	9.87 ± 0.00 ^d^	18.31 ± 0.05 ^a^	25	17
	ΣAAA ^7^	84.94 ± 1.59 ^c^	88.72 ± 0.05 ^b^	65.72 ± 0.06 ^f^	71.00 ± 1.10 ^e^	77.68 ± 0.42 ^d^	72.91 ± 0.81 ^e^	93.28 ± 0.20 ^a^	63	19
	ΣEAA/ TAA TAA	0.43 ± 0.00 ^c^	0.42 ± 0.00 ^c^	0.45 ± 0.00 ^a^	0.44 ± 0.00 ^b^	0.43 ± 0.00 ^c^	0.44 ± 0.00 ^b^	0.43 ± 0.00 ^c^		
	EAA/ NEAA	0.75 ± 0.00 ^c^	0.74 ± 0.00 ^c^	0.8 ± 0.01 ^a^	0.78 ± 0.01 ^b^	0.75 ± 0.01 ^c^	0.78 ± 0.01 ^b^	0.75 ± 0.00 ^c^		

Notes: ^1^ Total essential amino acids. ^2^ Total non-essential amino acids. ^3^ Total amino acids. ^4^ Hydrophobic amino acids, Gly + Ala + Pro + Val + Met + Ile + Leu + Phe. ^5^ Branched-chain amino acids, Val + Leu + Ile. ^6^ Sulphur amino acids, Met + Cys. ^7^ Aromatic amino acids, Tyr + Phe. Data is expressed as mean ± SD (*n* = 3); different superscript letters on same line refer to significant differences (*p* < 0.05).

**Table 3:** AAS values in 7 kinds of mulberry leaf glutelins.

Protein Samples							
	TSG	DSG	109G	C1G	C2G	C3G	C4G
Thr	1.66	1.88	1.37	1.48	1.70	1.46	1.91
Val	1.22	1.34	1.02	1.07	1.20	1.03	1.35
Ile	1.45	1.54	1.20	1.27	1.37	1.20	1.54
Leu	1.36	1.37	1.07	1.10	1.25	1.19	1.43
Lys	0.90	0.90	0.89	0.93	1.02	0.98	1.05
His	1.59	1.57	1.28	1.32	1.35	1.38	1.64
Met + Cys	0.78	0.78	0.52	0.58	0.65	0.45	0.83
Phe + Tyr	2.24	2.33	1.73	1.87	2.03	1.92	2.45
AAS	0.78	0.78	0.52	0.58	0.65	0.45	0.83

Note: The reference standard for AAS is the model recommended by FAO/WHO [36].

**Table 4:** Evolution of amino acid ratio coefficient method for 7 kinds of mulberry leaf glutelins.

Protein Source	Protein Characteristic Value	Recommended Composition of Essential Amino Acids of FAO/WHO							SRC	PDCAAS (%)
		Thr	Val	Ile	Leu	Lys	Met + Cys	Phe + Tyr		
TSG	RAA	1.12	1.36	1.55	1.21	0.70	0.69	1.35	70.70	66.40
	RCAA	0.98	1.19	1.36	1.06	0.61	0.60	1.18		
DSG	RAA	1.27	1.49	1.64	1.23	0.70	0.68	1.41	68.65	68.30
	RCAA	1.06	1.24	1.37	1.02	0.58	0.57	1.17		
109G	RAA	0.92	1.14	1.29	0.96	0.69	0.46	0.82	69.37	39.70
	RCAA	1.03	1.27	1.43	1.07	0.77	0.51	0.92		
C1G	RAA	1.00	1.20	1.36	0.98	0.72	0.51	0.92	70.49	37.00
	RCAA	1.05	1.25	1.42	1.03	0.76	0.53	0.96		
C2G	RAA	1.18	1.31	1.43	0.57	0.79	0.57	1.01	64.31	43.90
	RCAA	1.20	1.34	1.47	0.58	0.81	0.58	1.03		
C3G	RAA	0.99	1.15	1.29	1.06	0.76	0.39	0.91	68.77	32.80
	RCAA	1.06	1.23	1.37	1.13	0.81	0.42	0.97		
C4G	RAA	1.29	1.51	1.65	1.28	0.82	0.73	1.48	71.97	63.30
	RCAA	1.03	1.20	1.32	1.02	0.65	0.59	1.18		

**Table 5:** The solubilities of seven kinds of mulberry leaf glutelin and SPI (%).

pH	Protein Samples							
	SPI	TSG	DSG	109G	C1G	C2G	C3G	C4G
2	0.10 ± 0.00 ^e^	59.00 ± 6.61 ^a^	35.10 ± 0.11 ^b^	10.81 ± 1.67 ^d^	25.49 ± 4.86 ^c^	24.55 ± 1.69 ^c^	66.22 ± 3.41 ^a^	24.69 ± 2.15 ^c^
4	0.92 ± 0.28 ^c^	0.38 ± 0.14 ^c^	1.15 ± 0.45 ^c^	3.07 ± 0.08 ^c^	8.29 ± 2.82 ^b^	16.74 ± 2.57 ^a^	1.60 ± 0.30 ^c^	0.94 ± 0.29 ^c^
6	3.77 ± 0.71^e^	84.04 ± 4.05 ^a^	71.97 ± 5.91 ^b^	34.30 ± 2.31 ^d^	37.56 ± 6.56 ^d^	59.91 ± 2.48 ^a^	91.65 ± 1.72 ^a^	33.38 ± 6.88 ^d^
8	9.75 ± 1.56 ^e^	92.37 ± 3.12 ^a^	83.11 ± 4.91 ^bc^	64.34 ± 4.29 ^d^	84.89 ± 2.26 ^bc^	78.69 ± 0.15 ^c^	92.84 ± 2.33 ^a^	86.11 ± 2.08 ^ab^
10	34.03 ± 3.70 ^d^	92.89 ± 0.59 ^ab^	93.08 ± 2.67 ^ab^	82.92 ± 1.14 ^c^	94.86 ± 2.86 ^a^	87.13 ± 0.04 ^bc^	96.65 ± 2.37 ^a^	92.36 ± 0.86 ^a^
12	70.50 ± 5.77 ^c^	93.32 ± 5.78 ^a^	94.68 ± 3.17 ^a^	82.10 ± 7.00 ^b^	93.09 ± 2.95 ^a^	84.46 ± 2.75 ^ab^	94.82 ± 2.70 ^a^	84.35 ± 2.58 ^ab^

Data is expressed as the mean ± SD (*n* = 3); different superscript letters on the same line refer to significant differences (*p* < 0.05).

## Data Availability

The original contributions presented in this study are included in the article. Further inquiries can be directed to the corresponding authors.
